# Lower Diversity of Amylase-Trypsin Inhibitors and *Ex Vivo*-Released Opioid-Containing Peptides in Ancestral Compared to Modern Wheat Varieties Assessed by Proteomics and Peptidomics

**DOI:** 10.1021/acs.jafc.4c05959

**Published:** 2025-01-15

**Authors:** Tora Asledottir, Gianfranco Mamone, Gianluca Picariello, Gerd E. Vegarud, Arne Røseth, Pasquale Ferranti, Tove G. Devold

**Affiliations:** † Faculty of Chemistry, Biotechnology and Food Science, Norwegian University of Life Sciences, 1433 Ås, Norway; ‡ Institute of Food Science, National Research Council, 83100 Avellino, Italy; § Department of Internal Medicine, Lovisenberg Diaconal Hospital, 0456 Oslo, Norway; ∥ Department of Agriculture, University of Naples Federico II, 80055 Portici, Italy

**Keywords:** wheat, amylase-trypsin inhibitor, opioid peptides, *ex vivo* digestion, peptidomics

## Abstract

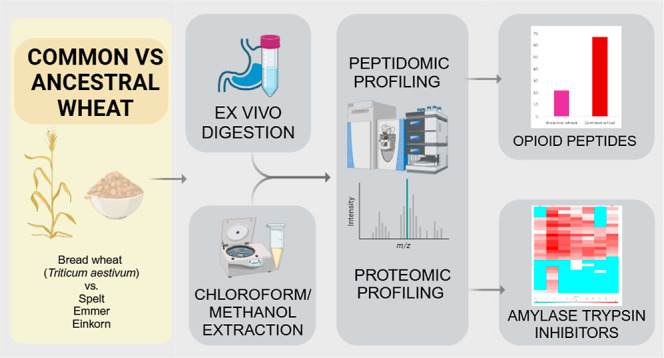

This study focused on identifying amylase-trypsin inhibitors (ATIs) in seven Norwegian-cultivated wheat varieties, including common wheat and ancestral species, and identifying potentially harmful opioid peptides within the *ex vivo* digesta of these wheats. LC–MS/MS analysis of tryptic peptides from ATI fractions revealed that the common wheat variety Børsum exhibited the highest diversity of ATIs (*n* = 24), while they were less represented in tetraploid emmer (*n* = 11). Hexaploid wheat Bastian showed low diversity and relative abundance of ATIs. Nevertheless, digestion of Mirakel and Bastian by human gastrointestinal juices released the highest number of opioid-containing peptides, representing both gluten exorphins and gliadorphin. In conclusion, emmer had the lowest levels of ATIs, while einkorn and spelt released the fewest opioid-containing peptides after *ex vivo* digestion. These results point to the potential lower harmful effects of ancestral wheat compared to common hexaploid wheat varieties for wheat-sensitive individuals.

## 1 Introduction

Nonceliac wheat sensitivity (NCWS) is a condition where individuals experience symptoms similar to those of celiac disease, such as abdominal pain, bloating, and fatigue, after consuming wheat products, despite not being diagnosed with celiac disease or a proven wheat allergy.^18^ There has been an increase in the prevalence of self-reported wheat-related intolerances over the past decade as on average 10% of individuals worldwide experience intolerance to various components within wheat, manifesting as a range of digestive disorders.^2^ The increase in NCWS is believed to be linked to the advancements in wheat breeding over the past century.^2^ Modern bread wheat varieties have been developed for climate adaptability, increased yield, improved resistance to plant diseases, enhanced protein content, and superior baking qualities compared to its predecessors. Despite these benefits, wheat-sensitive individuals have reported less severe or postponed reactions after consuming ancestral wheat varieties, such as emmer and einkorn, as opposed to modern ones. This observation implies that these ancestral species may have lower levels of components provoking wheat-related disorders.^26^

In common wheat (*Triticum aestivum*), amylase-trypsin inhibitors (ATIs) constitute up to 4% of total proteins (w/w).^6,10^ These ATIs include several proteoforms such as wheat ATI monomer 0.28, ATI homodimers 0.19 and 0.53, and ATI heterotetramers CM1, CM2, CM3, CM16, and CM17. The tetrameric inhibitors, soluble in chloroform/methanol (CM), are collectively known as CM proteins. Additionally, wheat contains trypsin-specific inhibitors referred to as CMX or WTI (wheat trypsin inhibitor) as well as amylase-subtilisin inhibitor (WASI) and chymotrypsin inhibitors (WCI and WCSI).^11^ In plants, ATIs serve as a defense mechanism against herbivores and parasites. However, as part of a diet containing wheat-based products, these inhibitors have shown to interfere with the activity of digestive enzymes, causing gastrointestinal (GI) symptoms in humans.^13^ ATIs are of interest due to their potential to exacerbate immune responses in individuals affected by either celiac disease or NCWS. Indeed, ATIs can trigger immune responses by activating Toll-like receptor 4 (TLR4), thereby promoting inflammatory conditions.^5,17^

Alongside ATIs, the gluten protein fraction of wheat harbors several amino acid sequences with structural motifs capable of binding to opioid receptors. Such opioid peptides share a tyrosine residue at the N terminal or at the penultimate position of the N terminal as a common structural element that underlies the binding to μ-opioid receptors with high affinity.^20^ As wheat proteins undergo hydrolysis during digestion, peptides with opioid-like properties are released, including exorphins and gliadorphins derived from glutenin and gliadin, respectively.^31^ These peptides are hypothesized to interact with opioid receptors within the human body, potentially inhibiting muscle contraction in the intestine and contributing to constipation, which can lead to digestive problems. Furthermore, these peptides are believed to have the potential to interfere with various physiological functions leading to neurological and behavioral symptoms in susceptible individuals.^21^ This activity is thought to stem from their ability to cross the blood brain barrier (BBB) and activate the central nervous system.^7^ However, it is important to note that no direct evidence currently supports this hypothesis, as studies have so far been limited to *in vitro* investigations.^32^ Opioid peptides may also undergo extensive first-pass metabolism in the liver before entering the systemic circulation, which can drastically reduce their bioavailability.^30^ Therefore, the role of these food-derived opioid peptides in intestinal disorders, particularly in the context of NCWS, remains largely speculative, and further research is required to better understand the mechanisms and significance of these peptides in such conditions.

Both wheat ATIs and opioid peptides have been associated with NCWS.^18,11^ This study aimed at identifying ATIs by a proteomic approach in seven Norwegian-cultivated wheat varieties, including the *T. aestivum* varieties Fram, Børsum, Bastian, and Mirakel and the ancestral wheat species spelt, emmer, and einkorn. In addition, wheat porridges of the same wheat types were subjected to *ex vivo* GI digestion with human gastric and intestinal juices for the peptidomic identification of potential opioids within the digesta.

## 2 Materials and Methods

### 2.1 Materials

Wheat samples were collected from an experimental field at NMBU Research Farm (Norwegian University of Life Sciences, Ås, Norway, 59°39′N 10°45′E) in 2017 and 2021. The wheat varieties were cultivated within the same field trial, in different plots measuring 4.5 m^2^ each, and were all spring types including the ancestral wheat species einkorn (diploid, AA, *Triticum monococcum*), emmer (tetraploid, AABB, *Triticum dicoccum*), and spelt (hexaploid, AABBDD, *Triticum spelta*), along with four varieties of common wheat (hexaploid AABBDD, *T. aestivum*) (Table 1). Each wheat type was collected from two different plots. Wheat harvested in 2017 was subjected to *ex vivo* digestion and opioid peptide profiling, and wheat harvested in 2021 was extracted and analyzed for ATIs. After harvesting, the wheat samples were subjected to drying at 30 °C for 3 days, reducing moisture levels to below 15%, prior to threshing and cleaning (Perten Instruments AB, Hägersten Sweden).

**Table 1:** Sample Overview with Genetic Background and Protein Content of the Wheat Types Studied. DW, Dry Weight

wheat type	species	genome	variety	protein g/100 g DW
Common wheat	*T. aestivum var. aestivum*	AABBDD	Mirakel	11.32
	*T. aestivum var. aestivum*	AABBDD	Bastian	13.18
	*T. aestivum var. aestivum*	AABBDD	Børsum	14.18
	*T. aestivum var. aestivum*	AABBDD	Fram	12.73
Spelt	*T. aestivum var. spelta*	AABBDD	Gotland	15.42
Emmer	*T. dicoccon*	AABB	Gotland	13.61
Einkorn	*T. monococcum*	AA	“unknown”	15.86

### 2.2 Processing of Wheat Samples

Einkorn, emmer, and spelt were dehulled manually after threshing. The samples were milled to whole grain flour by Falling Number Laboratory Mill 3100 with a 0.8 mm screen (Perten Instruments AB, Hägersten, Sweden) before characterization and processing. Wheat porridge was prepared for each wheat variety by mixing whole grain wheat flour and water (1:20 w/v). The mixture was heated at 100 °C in a water bath for 10–15 min with mixing by vortex every 2 min. Porridge samples were homogenized by Ultra-Turrax (T-18, IKA—Werke GmbH & Co. KG, Staufen, Germany) for 5 s, cooled, and stored at 4 °C until *ex vivo* digestion.

### 2.3 Characterization of Wheat Flour Composition

Analysis of the nitrogen and moisture contents of wheat was performed in duplicates per wheat sample. Total nitrogen of samples was determined by the Kjeldahl method on a Kjeltec 8400 (Foss, Hillerød, Denmark), and protein content was calculated using a 5.7 conversion factor. The moisture content of the wheat was determined by oven-drying milled samples for 24 h at 105 °C.

### 2.4 Extraction of ATIs from Wheat Flour

ATIs in milled wheat samples were extracted using chloroform/methanol extraction according to Sagu et al.^27^ with minor modifications. Whole grain milled wheat (100 mg) was defatted by mixing with petroleum ether (0.4 mL), and samples were shaken on ice for 10 min at 200 rpm before centrifugation at 10,000*g* for 5 min. The supernatant was discarded, chloroform/methanol (2:1) was added to the pellets, and samples were incubated on ice with shaking at 200 rpm for 10 min, before centrifugation as detailed above. The supernatants from four parallel extractions were collected, pooled, and dried under vacuum in a SpeedVac (Eppendorf concentrator plus, Hamburg, Germany) at 30 °C for approximately 2 h. The dried samples were redissolved in 0.4 mL of Tris-NaCl buffer at pH 7.1 and vortexed for 4 min, sonicated for 5 min, and centrifuged as described above. Finally, the supernatant was collected containing extracted ATIs. The extraction was performed in parallel, and protein content in extracts was determined using the micro-BCA protein assay according to the manufacturer’s instructions (Thermo Scientific, Waltham, MA, USA). The determination of protein content was performed in triplicate where mean amounts ±SEM was reported. Statistical significance (*p* < 0.05) between extracts was determined by one-way ANOVA, followed by Tukey’s test for post hoc pairwise comparisons (RStudio version 2024.09.1).

The extraction efficiency was evaluated by SDS-PAGE. In short, ATI extracts were mixed 1:1 with SDS buffer containing 62.5 mM Tris–HCl, pH 6.8, 2% SDS, 25% (v/v) glycerol, 0.01% bromophenol blue, and 200 mM DTT, and the mixture was heated at 95 °C for 5 min. 10 μL of samples was loaded onto the wells of a 12% Mini-PROTEAN TGX Stain-Free Precast Gel (Bio-Rad Laboratories Ltd., Hemel Hempstead, Herts, UK), and the gel was run at 200 V for 35 min. A low molecular mass protein ladder was used as a standard. Images were captured by a Gel Doc EZ Imager (Bio-Rad Laboratories Ltd., Hercules, CA, USA).

### 2.5 In-Solution Trypsinolysis and Solid-Phase Extraction of ATI Peptides

The ATI extracts (50 μL) were diluted in 50 mM ammonium bicarbonate (300 μL, pH 7.8) to a protein concentration of approximately 150 μg/mL, reduced by the addition of 25 μL of 1 M DL-dithiothreitol (DTT), and incubated for 30 min at 56 °C. For alkylation, 25 μL of 0.5 M iodoacetamide was added, and the samples were incubated in the dark at room temperature for 30 min. A 50 μL trypsin aliquot (10 μg/mL, 15,000 U/mg, Sequencing grade Modified Trypsin, Promega) was added to the samples and incubated overnight at 37 °C with shaking. After hydrolysis, tryptic peptides were acidified with 25 μL of 10% trifluoroacetic acid (TFA) and sonicated for 10 min. The peptides were extracted and purified by solid-phase extraction using the tips of the OMIX C18 pipet according to the manufacturer’s instructions (Agilent Bond Elut OMIX, Agilent Technologies, Palo Alto, CA, USA). After extraction, peptides were dried under vacuum at 30 °C in a SpeedVac for approximately 1 h and redissolved in 0.05% TFA and 2% acetonitrile (ACN) before analysis.

### 2.6 Identification of ATIs in Wheat Samples by UPLC–ESI–MS/MS

The extracted peptides were analyzed by using a nano-ultrahigh pressure liquid chromatography system (nanoElute, Bruker Daltonics, Bremen, Germany) linked to a timsTOF Pro mass spectrometer (Bruker Daltonics) equipped with a trapped ion mobility quadrupole time-of-flight detector. Peptide separation was achieved on a PepSep Reprosil C18 reverse-phase column (1.5 μm, 100 Å, 25 cm × 75 μm) connected to a ZDV Sprayer (Bruker Daltonics). The column temperature was maintained at 50 °C by using an integrated oven. Column equilibration was carried out under 800 bar pressure before loading samples. The flow rate was set at 300 nL/min, using a solvent gradient from 5% to 25% of solvent B over 70 min, followed by an increase to 37% over the next 9 min. The gradient then increased to 95% solvent B over 10 min and maintained at that level for an additional 10 min, totaling a 99 min runtime for peptide separation. Solvent A was 0.1% (v/v) formic acid in deionized water, and solvent B was 0.1% (v/v) formic acid in ACN.

The timsTOF Pro operated in positive ion mode using data-dependent acquisition PASEF with Compass HyStar software, version 5.1.8.1, and timsControl, version 1.1.19. The mass acquisition range was set from 100 to 1700 *m*/*z*. TIMS settings included a 1/*K*_0_ start of 0.85 V·s/cm^2^ and a 1/*K*_0_ end of 1.4 V·s/cm^2^, with a ramp time of 100 ms and a ramp rate of 9.42 Hz, and duty cycle 100%. The capillary voltage was adjusted to 1400 V, with a dry gas flow at 3.0 L/min and a dry temperature of 180 °C. MS/MS settings included 10 PASEF ramps per cycle, with a total cycle time of 0.53 s, a charge range from 0 to 5, a scheduling target intensity of 20,000, an intensity threshold of 2,500, active exclusion release after 0.4 min, and CID collision energy ranging from 27 to 45 eV. The mass spectrometry proteomics data have been deposited to the ProteomeXchange Consortium via the PRIDE partner repository with the data set identifier PXD059081.^25^

The acquired spectra were submitted to MaxQuant software (version 2.6.5) with default settings and label-free quantification (LFQ) enabled.^4^ The data was searched against the proteome of *Triticum* (downloaded from UniProt, November 2024). The search parameters were set as follows: oxidation and acetyl as variable modification with a first search peptide tolerance of 20 ppm and a main search error of 4.5 ppm. Carbamidomethyl of cysteine was set as fixed modifications, trypsin was selected as specific digestion mode, and maximum two missed cleavages were allowed. Maximum peptide mass was set at 4600 Da, and a protein false discovery rate of 0.01 was used. Proteins identified by MaxQuant were processed using Perseus software^35^ to elucidate differences across wheat samples. The data set was filtered to remove proteins only identified by site, potential contamination, and reverse hits. LFQ intensities were log2-transformed, and missing values were replaced with a fixed value of 0. ATI-identified proteins were selected, and a heat map was generated, with minimum and maximum intensity values represented by blue and red colors, respectively.

### 2.7 *Ex Vivo* GI Model Digestion of Wheat Porridge

The *ex vivo* digestion of wheat porridge, using human GI digestive juices under conditions of the standardized INFOGEST consensus static digestion model,^3^ was performed as described previously.^1^ Human gastric and duodenal juices were collected through aspiration of healthy volunteers (*n* = 20) aged 20–41 at Lovisenberg Diaconal Hospital, Norway, according to Ulleberg et al.^36^ The aspirates were pooled from all volunteers and stored at −20 °C for 24 h and then at −80 °C until use. All volunteers gave their informed consent for inclusion before participation, and the aspiration procedure was approved by the Norwegian Regional Committees for Medical and Health Research Ethics. The *ex vivo* digestion was conducted with approximately 1 g of porridge (5 mg/mL protein) through the oral phase, using salivary-simulated fluids containing human α-amylase (75 U/mL, Sigma), gastric phase containing human gastric juice corresponding to pepsin activity of 2000 U/mL, and intestinal phase containing human duodenal juice corresponding to trypsin activity of 100 U/mL. End-point sampling of digesta was performed after a total 2 h gastric and 2 h duodenal digestion. Inhibition of protease activities in the *ex vivo* digesta was achieved with the addition of 5 mM Pefabloc (Sigma-Aldrich, St. Louis, MO, US). The *ex vivo* digestion was performed in parallel, and all samples were immediately stored at −20 °C until peptidomic analysis.

### 2.8 Peptidomic Identification of Wheat Opioid Peptides in Digesta by UPLC–ESI–MS/MS

*Ex vivo*-digested wheat porridge samples were desalted and eluted as described in Asledottir et al.^1^ MS analysis of peptides was performed using a Q Exactive Orbitrap mass spectrometer (Thermo Scientific, San Jose, CA, USA), online coupled with an Ultimate 3000 ultrahigh-performance liquid chromatography instrument (Thermo Scientific, San Jose, CA, USA). Peptides were loaded through a 20 mm long × 100 μm internal diameter precolumn (LC Packings, San Jose, CA, USA) and separated by an EASY-Spray PepMap C18 column (2 μm, 25 cm to 75 μm, 3 μm particles, 100 Å pore size, Thermo Scientific, San Jose, CA, USA). Eluent solutions and all operating parameters of the MS are detailed in Asledottir et al.^1^ Peptides were identified using Proteome Discover 2.1 software based on the Sequest searching algorithm taxonomically restricting the search to the *Triticum* database (extracted from UniProt in February 2020). Peptide identification was validated at a false discovery rate of 0.01. The peptide list retrieved was manually searched for identification of known opioids within the digesta, based on sequences listed in the work of Liu and Udenigwe^21^ and Garg et al.^8^

## 3 Results and Discussion

### 3.1 Gross Composition of Raw Materials

All wheat samples were subjected to protein determination, and their values are listed in Table 1. The protein content varied among the wheat types, with common wheat types (*T. aestivum*) ranging from 11.32 g/100 g DW (Mirakel) to 14.18 g/100 g DW (Børsum). In contrast, the ancestral wheat types, including einkorn (15.86 g/100 g of DW), emmer (13.61 g/100 g of DW), and spelt (15.42 g/100 g of DW), exhibited higher protein contents. These findings align with those of Geisslitz et al.,^9^ who reported higher total protein content in ancestral wheat compared to common wheat. Notably, their study, which analyzed wheat grown at four different locations in Germany, highlighted an equally significant influence of growth location and wheat species on the protein content.

### 3.2 Identification and Relative Quantification of ATIs in Wheat Samples and *Ex Vivo* Digesta

**Figure 1:**
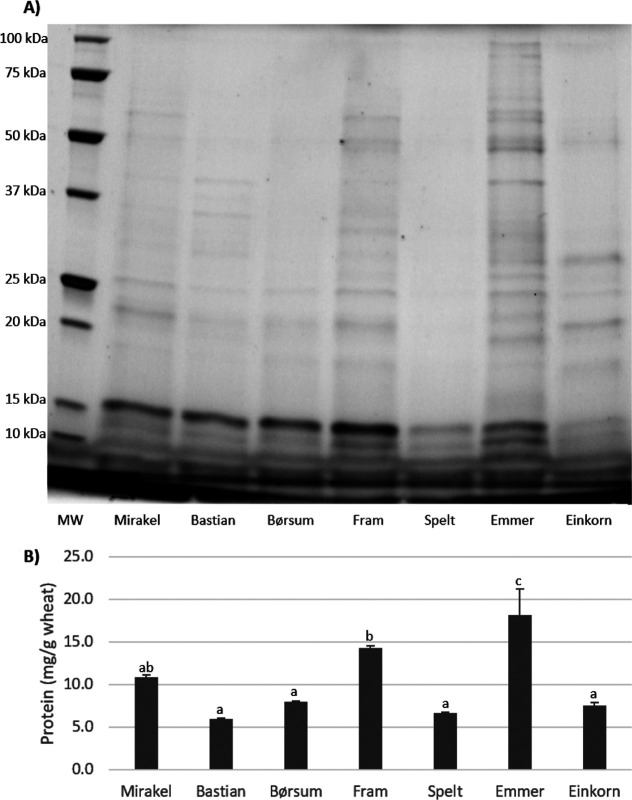
Chloroform/methanol extracts from seven Norwegian-cultivated wheat varieties. Extracts were (A) separated by SDS-PAGE and (B) measured for protein concentration by micro-BCA protein assay. Protein concentrations are plotted as means with error bars (SEM, *n* = 3). Statistically significant differences at *p* < 0.05 are denoted by different letters. MW, molecular weight standard.

SDS-PAGE separation of ATIs, purified based on the chloroform/methanol solubility method, is illustrated in Figure 1A. The protein concentrations in the extracts, presented in Figure 1B, showed significant variation. Emmer had the highest concentration (∼18 mg/g of wheat), followed by Fram (∼14 mg/g of wheat). Mirakel displayed intermediate levels similar to Fram but higher than the other wheat types. Bastian, Børsum, spelt, and einkorn had the lowest concentrations (6–8 mg/g wheat), with no significant differences among them. The CM extracts of common wheat (Mirakel, Bastian, Børsum, and Fram) displayed the most intense bands around 15 kDa, while spelt and einkorn exhibited weaker bands, indicating lower relative content of ATIs, consistent with protein concentration measurements of the extracts. Overall, the SDS-PAGE results showed that the low molecular weight proteins were effectively extracted and purified, aligning with the findings of Sagu et al.^28^

**Figure 2:**
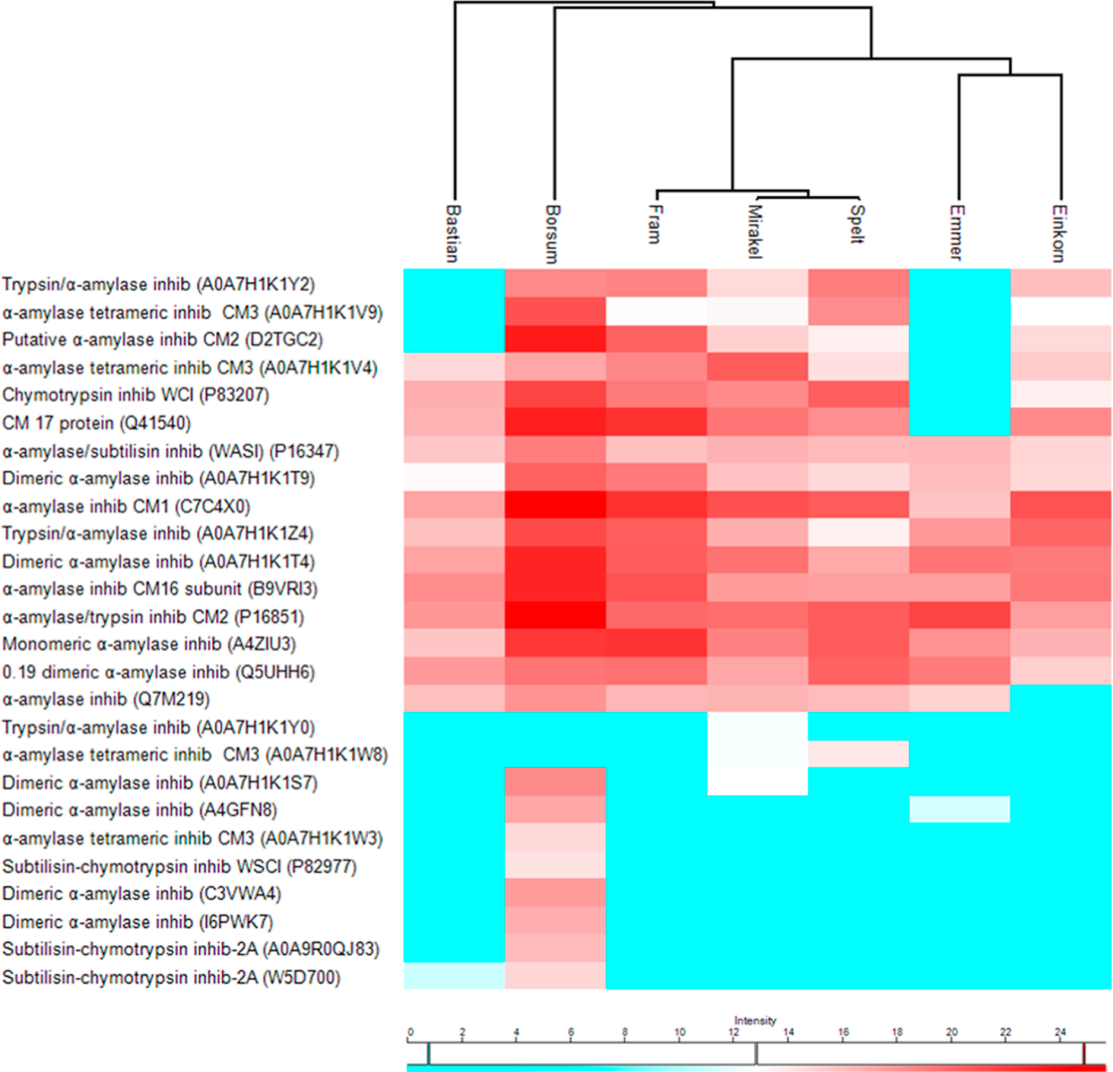
Heat map of UPLC–ESI–MS/MS-based LFQ of ATIs in low-molecular weight protein extracts from seven wheat varieties. Columns represent wheat varieties and rows; 26 different ATIs identified. The accession number listed in parentheses refers to the leading razor protein entry. High and low intensity values are indicated by red and blue colors, respectively.

Furthermore, extracted proteins were trypsinized and subjected to LC–MS/MS analysis for the identification of ATIs in wheat samples. In total, after homology filtering, 133–269 different proteins were identified from each individual wheat extract (data not shown). The ATI entries were selected manually, and they are represented in the heat map of Figure 2 along with their intensity inferred from MS-based LFQ data (Supplementary Table S1). In total, 26 different ATIs were identified. The most recurrent and relatively abundant ATIs were CM1 (UniProt KB accession number P16850) and CM2 (P16851). ATI CM16 (P16159), monomeric ATI 0.28 (P01083), endogenous α-amylase/subtilisin inhibitor (WASI) (P16347), and dimeric ATIs 0.19 (P01085 and Q5UHH6) were identified in all wheat samples. ATI CM3 (A0A7H1K1V4) and CM17 (Q41540) were only lacking in emmer, while CMx (A0A7H1K1Y2) was missing in emmer and Bastian. The ATI UniProt KB Q7M219 sharing high homology with ATI CM3 was identified in all of the wheat varieties but einkorn.

Overall, several additional inhibitors were identified in Børsum including subtilisin-chymotrypsin inhibitor and xylanase inhibitor, which can also bind and inhibit barley α-amylase.^16^ Børsum showed the highest diversity (*n* = 24) and abundance of ATIs, followed by Mirakel (*n* = 19) and Fram (*n* = 16). Diversity and overall abundance of ATIs in spelt (*n* = 17) was comparable to other hexaploid wheat. In contrast, hexaploid wheat Bastian was relatively low in ATIs (*n* = 14) (Figure 2, Supplementary Table S1). ATIs were less represented in emmer and einkorn than in hexaploid wheat varieties, although several major ATIs were detected. The subunits of the tetrameric inhibitors CM3 and CM16 are encoded by genes on different chromosomes, including loci on the A genome, which explains the identification of some of these ATIs in the ancestral diploid wheat einkorn (AA genome). Geisslitz et al.^10^ also reported few ATIs identified in the ancestral wheat einkorn, in addition to low content of these, compared to other species (common wheat, durum, spelt, and emmer). In agreement with these authors, CM17 was missing in tetraploid emmer, while 0.19 and CM1 occurred at low abundance. The mentioned study investigated eight different cultivars of each wheat grown at three different locations, and the inhibitors 0.19, CM3, and WTI were only detectable in three, six, and 11 out of the 24 einkorn samples, respectively (compared to being detected in all 24 samples of common wheat). The authors concluded that the ancient wheat species einkorn, emmer, and spelt represent promising candidates in further breeding for reduced ATI content. Moreover, einkorn ATI sequences are substantially different from those of hexaploid wheat, showing more extensive degradation during *in vitro* digestion and lower activation of the innate immune response in celiac disease, measured by IL-8 and TNF-α mRNA levels.^15^ In fact, the inhibitors CM3 and 0.19 have shown to be the most potent ATI activators of TLR4 and highly resistant to intestinal proteolysis.^37^ However, the correlation between bioactivity and ATI concentration remains to be established.^10,29^

Geisslitz et al.^12^ highlighted the reliability of high-resolution MS for ATI quantification, emphasizing the importance of peptide selection and software on the results. LFQ performed with high-resolution MS data, like our approach, provided reliable identification and quantification comparable to labeled quantification methods. In our study, we analyzed chloroform–methanol (CM) protein extracts. While we successfully identified all major ATIs, additional extraction methods using chaotropic agents could expand the protein inventory and improve the accuracy of the relative quantification.

Additionally, the *ex vivo* digesta of wheat porridge were screened for ATI-derived peptides. Interestingly, very few ATI-derived peptides were found in the digesta, namely, two in einkorn and 4–8 peptides each in emmer and common hexaploid wheat digesta. This suggests a high degree of hydrolysis of ATIs during *ex vivo* digestion. Our study performed end-point sampling of digesta (i.e., after 4 h GI digestion). Therefore, degradation of ATIs into undetectable fragments is a possibility as the applied proteomic method was unable to identify peptides shorter than five amino acids. Einkorn ATIs have shown higher susceptibility to enzymatic hydrolysis compared to common wheat varieties, where the ancestral wheat displayed a lower number of detectable ATI-derived peptides during digestion, which was also supported by their reduced ability to trigger innate immunity in celiac disease.^15^

### 3.3 Identification of Opioid Peptides in *Ex Vivo*-Digested Wheat Porridge

Peptides within the digesta of different wheat varieties were identified through a UPLC–ESI–MS/MS-based peptidomic approach, which generated a list of nonredundant unique peptides. In total, 1157 unique peptides were identified within the digesta. The lowest number of unique peptides identified was from digested spelt (*n* = 178), and the highest number was from digestion of the modern wheat variety Mirakel (*n* = 450) (Table 2).

**Table 2:** Total Number of Peptides, Unique Peptides, and Opioid-Containing Peptides Identified in Wheat Digesta

wheat type	number of peptides in digesta	unique peptides identified	opioid-containing peptides	percentage opioids/unique
Mirakel	2689	450	26	6
Bastian	2472	424	24	6
Børsum	1641	290	10	3
Fram	1335	235	7	3
Spelt	1051	178	5	3
Emmer	1551	263	11	4
Einkorn	1586	215	6	3

**Figure 3:**
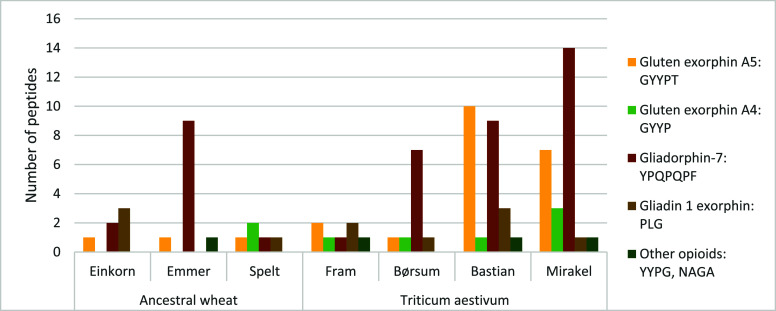
Frequency of opioid-containing peptides identified in *ex vivo* porridge digesta of seven Norwegian-cultivated wheat varieties. Colors of bars represent specific opioid sequences listed in the right panel.

While the free opioid sequences as listed in the work of Liu and Udenigwe^21^ and Garg et al.^8^ were not detected in any of the wheat varieties, the digesta contained peptide precursors encrypting the opioid motifs. The peptide list was used to manually search for opioid-containing peptides matching the sequences of gluten exorphins, gliadorphins, and other previously reported wheat opioids.^8^ Einkorn and spelt produced fewest opioid-containing peptides, namely, 6 and 5, respectively, representing both exorphins and gliadorphins. The hexaploid wheat Fram released seven opioid-containing peptides, followed by Børsum (*n* = 10), emmer (*n* = 11), Bastian (*n* = 24), and Mirakel (*n* = 26). Figure 3 illustrates the frequency of different opioid precursor peptides in wheat porridge digesta. The most recurrent opioid sequence was gliadorphin-7, YPQPQPF, which was found within 14 different peptides in Mirakel digesta and within 9 different peptides in Bastian and emmer digesta. Derived from α-gliadin, this opioid sequence has been shown to interact with opioid-like receptors on human peripheral blood mononuclear cells, with its activity inhibited by naloxone, suggesting that its effects are mediated through opioid receptor signaling pathways.^14,24^. Trivedi et al.^34^ also demonstrated that this peptide activates opioid receptors, in addition to regulating cysteine uptake in GI and neuronal cells. Furthermore, they explored the peptide’s potential to exert antioxidant and epigenetic changes by influencing cellular redox status and methylation capability.

The opioid sequence of gluten exorphin A5 (GYYPT) released from high molecular weight glutenins was detected in 10 and 7 different peptides from Bastian and Mirakel digesta, respectively. Stuknytė et al.^31^ investigated the transport of this peptide across monolayers of Caco-2/HT-29 coculture, after their identification in the *in vitro* digesta of bread and pasta. They noted that only 3% of the peptide introduced apically remained intact in the basolateral compartment of the absorption model, indicating a high susceptibility to enzymatic degradation by peptidases and a significant reduction in bioavailability during intestinal absorption. However, exogenous food-derived opioid peptides may exhibit preabsorptive activities when binding opioid receptors in the GI tract, affecting intestinal functions.^23,33^

Landolfi et al.^19^ investigated the peptide profile of *in vitro*-digested modern and hybrid wheat in relation to nitrogen fertilization. Their findings highlighted gluten exorphin A5 as the most frequently occurring opioid sequence across all wheat types analyzed. The number of peptides identified (4–12 precursor peptides) was consistent with our findings. Notably, gluten exorphin A4 (GYYP) which was absent in einkorn and emmer in our study was also missing in the digesta of the hybrid wheat type tritordeum. The absence of this opioid sequence in einkorn, emmer, and tritordeum may be partially attributed to the lack of the D genome in these wheat types. Interestingly, gliadorphin-7 was detected in only 1–3 peptides within the wheat digesta analyzed by Landolfi et al.^19^

It is important to emphasize that the presence of opioid peptides in wheat and their role in wheat sensitivity reactions are complex topics that require further investigation. Significant knowledge gaps remain concerning food-derived opioid peptides and how their effects manifest in individuals. The scientific community is actively studying these peptides and their potential impact on human health to gain a clearer understanding of their significance in wheat-related sensitivities and disorders.

Further research should focus on monitoring the release of opioid-active sequences from peptide precursors during digestion, particularly through the action of exo- and endopeptidases of the intestinal brush border membrane.^22^ Additionally, it is important to investigate the implications for individuals with impaired intestinal barrier function and to assess the degree to which these peptides are absorbed into the bloodstream. While it has been hypothesized that these peptides may cross the BBB and activate the central nervous system, no direct evidence supports this as current findings are limited to *in vitro* studies.

MS-based bottom-up proteomics uncovered 26 different α-amylase and -trypsin inhibitors across seven Norwegian-cultivated wheat varieties. The modern wheat variety Børsum exhibited the highest diversity and abundance of ATIs (*n* = 24), while the tetraploid wheat variety emmer displayed the lowest ATI levels (*n* = 11). *Ex vivo* digestion of Mirakel and Bastian released the highest number of opioid-containing peptides, including both gluten exorphins and gliadorphins, whereas einkorn and spelt digesta contained the fewest. In conclusion, our findings suggest that ancestral wheat varieties, particularly einkorn and emmer, may have lower levels of potentially harmful ATIs and opioid-containing peptides compared with modern hexaploid wheat varieties, offering potential benefits for wheat-sensitive individuals. These findings highlight the potential advantages of advocating for the increased use of ancestral wheat among individuals with wheat sensitivity. This study shows the importance of considering wheat components beyond gluten in the context of digestive disorders, and dedicated future research should focus on studying improved tolerance of selected wheat varieties in wheat-sensitive individuals through clinical trials, preferably including fermentation strategies of wheat products, such as sourdough, for enhanced degradation.

## Supporting Information Available

List of all ATIs identified with corresponding intensities, retrieved through LFQ of MS-based proteomics (XLSX)

## Notes

For aspiration of gastric and duodenal juices, all subjects gave their informed consent for inclusion before participation. The aspiration procedure was approved by the Regional Committees for Medical and Health Research Ethics (REK 2012/2230 and 2012/2210) in Norway.
